# Ocular manifestations in Chinese adult patients with NLRP3-associated autoinflammatory disease

**DOI:** 10.1038/s41598-021-91315-y

**Published:** 2021-06-07

**Authors:** Tianli Meng, Di Wu, Yi Luo, Na Wu, Mengzhu Zhao, Min Shen, Weihong Yu

**Affiliations:** 1 Department of Rheumatology, National Clinical Research Center for Dermatologic and Immunologic Diseases (NCRC-DID), Key Laboratory of Rheumatology and Clinical Immunology, Ministry of Education, Peking Union Medical College Hospital, Chinese Academy of Medical Sciences & Peking Union Medical College, No. 1 Shuaifuyuan, Dongcheng District, Beijing, 100730 China; 2 Department of Rheumatology, Jilin Province People’s Hospital, Changchun, China; 3 Department of Ophthalmology, Key Laboratory of Ocular Fundus Diseases, Peking Union Medical College Hospital, Chinese Academy of Medical Sciences & Peking Union Medical College, No. 1 Shuaifuyuan, Dongcheng District, Beijing, 100730 China

**Keywords:** Rheumatic diseases, Autoinflammatory syndrome

## Abstract

*NLRP3*-associated autoinflammatory disease (*NLRP3*-AID) is a rare autosomal dominant disorder involving multiple systems. We aim to assess the ocular manifestations of Chinese adult patients with *NLRP3*-AID. Twelve adult patients (> 18 years old) were diagnosed as *NLRP3*-AID at the Department of Rheumatology, Peking Union Medical College Hospital. All patients underwent ophthalmologic evaluation by an ophthalmologist. Clinical and genetic features of these patients were collected and compared with those from Caucasian population. A total of 7 *NLRP3*-AID patients (58%) 14 eyes had ocular manifestations. Five *NLRP3* variants were identified, and 3 patients (43%) with severe ocular damages were all found to have the *NLRP3* T348M variant. The incidences of papilledema and optic atrophy in the Chinese adult *NLRP3*-AID patients of moderate type were similar to those in the Caucasian *NLRP3*-AID patients of severe type. This is the first cohort of Chinese adult *NLRP3*-AID patients with ocular involvement. Ocular manifestations were diverse and even severe in *NLRP3*-AID, particularly in patients with the moderate phenotype, and may have relationship with genotypes. Awareness of these manifestations by rheumatologists and ophthalmologists could help to avoid the irreversible ocular damages.

## Introduction

Systemic autoinflammatory diseases (SAIDs) are a group of inherited disorders, which are usually caused by dysregulation of innate immunity leading to cytokines overproduction, and are lack of autoantibodies with high titer or antigen-specific T lymphocytes. Recurrent fever is the most common feature of SAIDs, whereas a wide range of inflammatory manifestations are variously combined with it, involving the skin, joints, muscle, gastrointestinal tract, ears, and central nervous system^1,2^. Inflammatory ocular involvement was frequently reported in Blau syndrome^3,4^, however, it may also be presented in the other SAIDs and some are even serious. In recent years, the inflammatory eye involvement has been increasingly explored in patients with *NLRP3*-associated autoinflammatory disease (*NLRP3*-AID) (formerly called cryopyrin-associated periodic syndrome, CAPS)^5^.

*NLRP3*-AID is an autosomal dominantly inherited SAID caused by mutations in the NLR family pyrin domain containing-3 (*NLRP3*) gene on chromosome 1q44, leading to enhanced activation of the NLRP3-inflammasome and overproduction of interleukin (IL)-1β^6,7^. It includes three overlapping clinical entities of increasing disease severity: the mild (familial cold autoinflammatory syndrome, FCAS), moderate (Muckle–Wells syndrome, MWS), and severe phenotypes (chronic infantile neurological cutaneous and articular syndrome, CINCA). *NLRP3*-AID is characterized by recurrent fever, urticaria-like rash and non-infectious inflammation in central nervous system, accompanied by arthritis/arthralgia and sensorineural deafness^8^. In regard to the ocular involvement, chronic conjunctivitis is known as the most frequent in *NLRP3*-AID, and serious involvement of eyes can be seen in the severe phenotype and leads to bad sequelae^9^. Hence, both rheumatologists and ophthalmologists should be aware of ocular manifestations related to *NLRP3*-AID. To the best of our knowledge, there have been no reports about the ocular manifestations in Chinese adult patients with *NLRP3*-AID.

Our tertiary medical center has the only adult SAIDs center in China. We have recently reported Chinese adult patients with *NLRP3*-AID who showed different phenotypes and genotypes compared with Caucasians and children with *NLRP3*-AID^10,11^. Herein, we described the ocular manifestations of adult *NLRP3*-AID in the Chinese population and compared them to the Caucasian cohorts of *NLRP3*-AID.

## Patients and methods

A total of 12 adult patients (> 18 years of age) were diagnosed as *NLRP3*-AID at the Department of Rheumatology, Peking Union Medical College Hospital, from April 2015 to January 2020. Demographic data, clinical characteristics, and laboratory tests were collected. All *NLRP3*-AID patients underwent ophthalmologic evaluation by an ophthalmologist. Past ocular medical record and diagnosis was carefully reviewed if the patient had prior consultations in other eye clinics. Mild ocular manifestations were defined as conjunctivitis, uveitis, and papilledema, while severe ocular involvement included optic neuritis, optic nerve atrophy, cataract, and glaucoma^12^. Whole exome sequencing by Next Generation Sequencing was performed in each patient (Joy Orient Translational Medicine Research Centre Co., Ltd, Beijing, China). This research was approved by the Institutional Review Board of Peking Union Medical College Hospital and performed according to the Declaration of Helsinki. Informed consent for both study participation and publication of identifying information/images in an online open-access publication (when applicable) was obtained from each participant^11^.

The ocular manifestations of Chinese adult patients with *NLRP3*-AID were compared with those of patients from other countries, by using the data from two publications with large cohorts of patients with *NLRP3*-AID^12,13^.

Demographic results were expressed as median and range. Chi-square or Fisher’s exact test was used for comparing frequencies of clinical manifestations between Chinese adult *NLRP3*-AID patients with and without ocular involvement^12^.

## Results

### Demographic data

**Figure 1:**
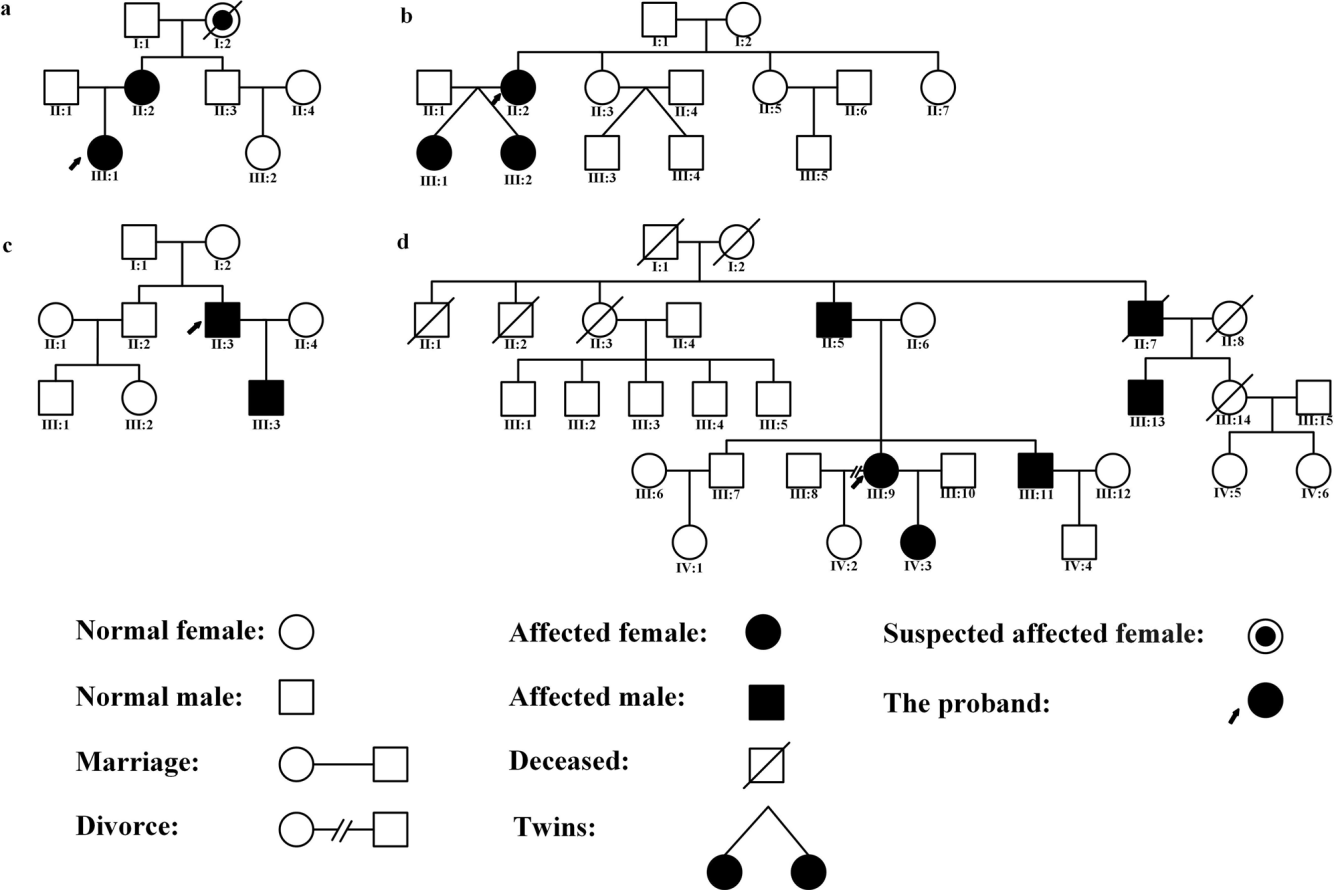
The pedigree charts of patients 3 (**a**), 5 (**b**), 6 (**c**), and 7 (**d**).

All of the 12 adult patients were of moderate phenotypes of *NLRP3*-AID (MWS). They were all Chinese Han nationality. Among them, 7 patients (58%) had eye manifestations. The overall ratio of male to female was 4:3. The median age at the time of diagnosis was 31 years old (range 20–45), and the median age of disease onset was 2 years old (range 0–10). The median time from disease onset to diagnosis was 26 years (range 12–39). Four out of seven patients (57%) reported positive family histories (Fig. 1).

### Phenotypes and genotypes of *NLRP3*-AID patients with ocular involvement

All of the 7 *NLRP3*-AID patients had periodic fever (100%), mostly moderate to high fever, among whom 5 (71%) were triggered by cold. Each febrile episode lasted several hours to several days, once every several weeks to months. All patients (100%) presented skin rash, with 5 (71%) manifested as urticaria, 2 (29%) erythema nodosa and 2 (29%) erythema (29%), distributed in the face, trunk and limbs. Arthralgia/arthritis, hearing loss and central nervous system involvements were the most prevalent features, each of which observed in 6 (86%) patients. As to the central nervous system, symptoms included headache (5, 71%), vertigo (3, 43%), aseptic meningitis (3, 43%), intracranial hypertension (3, 43%), nausea and vomiting (2, 29%), epileptic seizures (1, 14%), brain atrophy (1, 14%) and hydrocephalus (1, 14%). Myalgia (4, 57%), swelling of the lower extremities (2, 29%), oral ulcers (2, 29%), enlarged lymph nodes (2, 29%), and abdominal pain (1, 14%) could also be seen. No patients had amyloidosis. Acute phase reactants increased during the disease attacks, and decreased in the intervals in all patients, but couldn’t be normal in some of them. Five heterozygous variations of the *NLRP3* gene (NM_001243133.1) were identified in these 7 patients: T348M (exon3, c.1043C>T, rs151344629) (3, 43%), V70M (exon1, c.208G>A, rs117287351) (1, 14%), D303G (exon3, c.908A>G, rs180177447) (1, 14%), G326E (exon3, c.977G>A, rs180177456) (1, 14%) and A439V (exon3, c.1316C>T, rs121908146) (1, 14%) (Table 1).

**Table 1:** Clinical and genetic features of 7 *NLRP3*-AID patients with ocular involvement.

Patients	1	2	3	4	5	6	7
Gender	M	M	F	M	F	M	F
Ethnicity	Han	Han	Han	Han	Han	Han	Han
Age at onset, years old	2	2	0 (at birth)	10	2	6	6
Age at diagnosis, years old	23	31	20	22	39	32	45
Diagnosis delay, years	21	29	20	12	37	26	39
Family history	−	−	+	−	+	+	+
Fever	+	+	+	+	+	+	+
Rash	+	+	+	+	+	+	+
Oral ulcers	+	−	−	−	−	−	+
Arthralgia/arthritis	−	+	+	+	+	+	+
Hearing loss	−	+	+	+	+	+	+
Central nervous system involvement	+	+	−	+	+	+	+
Lymphadenopathy	−	+	−	−	+	−	−
Lower limbs edema	−	−	−	−	+	−	+
Myalgia	+	−	−	+	−	+	+
Abdominal pain	−	−	−	−	−	−	+
Fatigue	+	+	+	+	+	+	+
Amyloidosis	−	−	−	−	−	−	−
Eye involvement							
Conjunctivitis	+	+	+	+	+	+	+
Uveitis	−	−	+	−	−	−	+
Glaucoma	−	−	−	−	−	−	+
Papilledema	−	+	+	+	+	+	−
Optic neuritis	−	+	−	−	+	−	−
Optic atrophy	−	+	−	+	−	−	−
Acute phase reactants elevation	+	+	+	+	+	+	+
*NLRP3* variants	p.V70M	p.T348M	p.D303G	p.T348M	p.T348M	p.G326E	p.A439V
Treatments*							
Glucocorticoids	+++^$^	++	NU	++	++	NU	++
Immunosuppressants	NU	MTX, ++	MTX, ++	MTX, ++	MMF, ++	NU	AZA,++
TNFα inhibitors	NU	+++	+++	+++	NU	NU	NU

*MTX* methotrexate, *MMF* mycophenolate mofetil, *AZA* azathioprine, *NU* not used.

*−poor response; +mild response; ++moderate response; +++good response. ^$^Oral treatment only during attacks.

**Figure 2:**
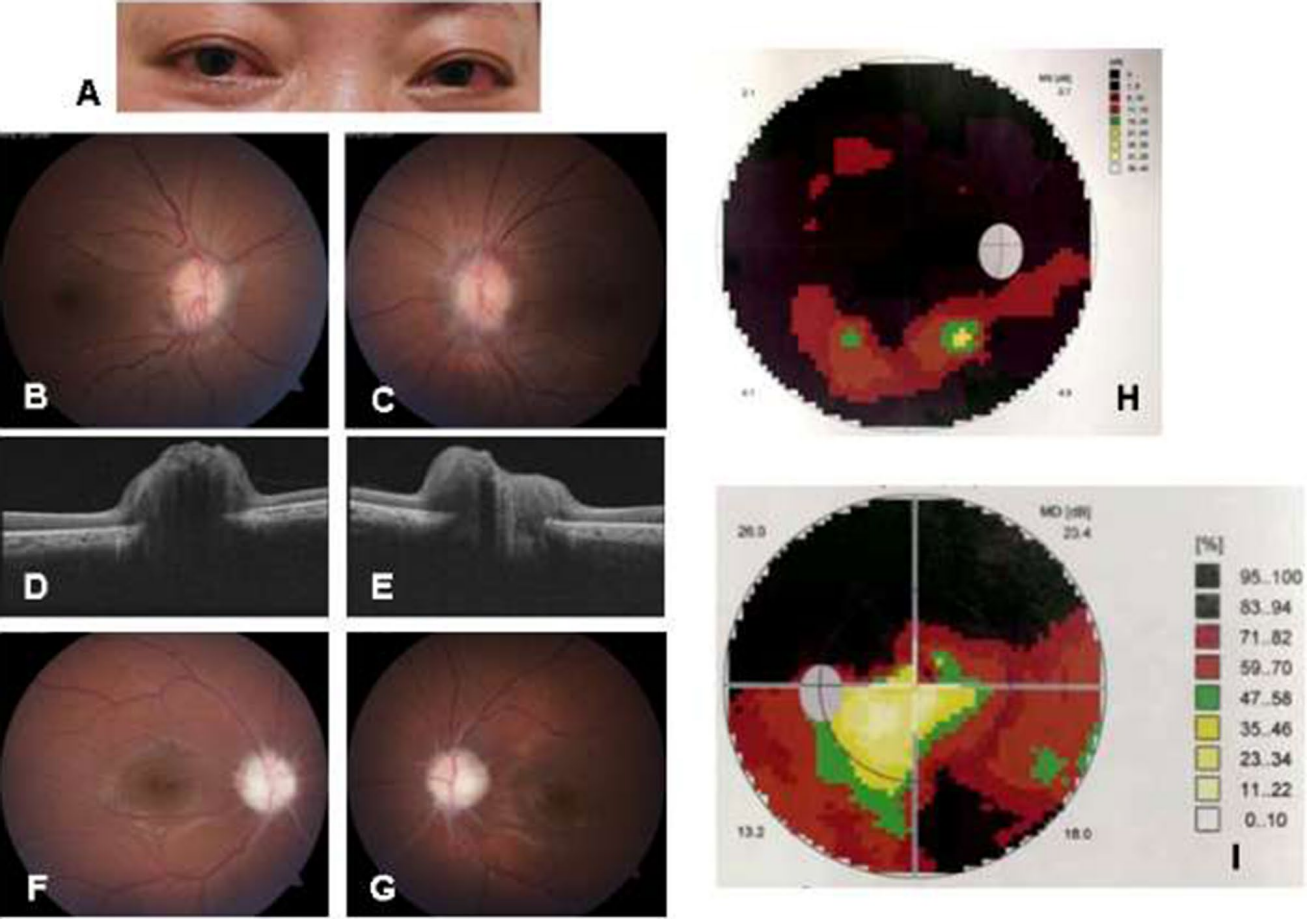
Ocular manifestations of Chinese adult *NLRP3*-AID patients. (**A**) Chronic conjunctivitis (patient 5). (**B**, **C**) Fundus photography (FP) showed optic disc swelling with blurred margin indicating bilateral papilledema (right eye and left eye, respectively) (patients 6). (**D**, **E**) Optical coherence tomography (OCT) examination confirmed the presence of bilateral papilledema (right eye and left eye, respectively) (patients 6). (**F**, **G**) FP demonstrated bilateral papilledema and pale-colored optic disc indicating optic atrophy (right eye and left eye, respectively) (patient 4). (**H**, **I**) Perimetry examination showed bilateral constriction of visual field consistent with optic atrophy (patient 4).

The median age of the first ocular symptoms presented was 18 years old (range 2–41), with 2 (29%) appeared in the early stage of disease. The initial ocular signs were usually reported as red eyes (6, 86%) and blurred vision (2, 29%). Among these 7 patients, 14 eyes had ocular manifestations with bilateral involvement with same diagnosis. Conjunctivitis was the most common form of ocular involvement (7, 100%), while uveitis was found in 2 patients (29%), and glaucoma in 1 (14%). No patients were diagnosed with keratitis. Five patients (71%) had papilledema. Two patients (29%) had optic neuritis, and 2 (29%) had optic atrophy (Fig. 2). Patients (3, 43%) who had severe ocular manifestations as optic neuritis and optic atrophy were all found to have an *NLRP3* T348M variant.

In regard to the treatments, one patient with mild symptoms was well controlled by taking prednisone only during the disease attacks. The other 4 patients who were given glucocorticoids and immunosuppressants such as methotrexate and azathioprine, showed moderate responses but had problems with steroids tapering. In our study, 3 patients (43%) had good responses to Etanercept (Table 1).

### Comparison of ocular manifestations in *NLRP3*-AID between the Chinese and other populations (Table 2)

**Table 2:** Comparison of phenotypic features among reported cohorts.

Characteristics	Our study (n = 12)	Levy et al.^12^ (n = 136)	Dollfus et al.^13^ (n = 31)
Male: female ratio	1:1	69:67	9:22
Origin (%)	Chinese (100)	Caucasians (100)	Caucasians (100)
Phenotype of *NLRP3*-AID (%)	Moderate (MWS) (100)	*NLRP3*-AID*	Severe (CINCA) (100)
Eye involvement (%)	7 (58)	97 (71)	31 (100)
Conjunctivitis (%)	7 (58)	87 (64)	20 (65)
Uveitis (%)	2 (17)	9 (7)	16 (52)
Keratitis (%)	0 (0)	0 (0)	13 (42)
Vitritis (%)	0 (0)	0 (0)	4 (13)
Cataract (%)	0 (0)	4 (3)	5 (16)
Glaucoma (%)	1 (8)	2 (1)	0 (0)
Papilledema (%)	5 (42)	29 (21)	13 (42)
Optic neuritis (%)	2 (17)	0 (0)	0 (0)
Optic atrophy (%)	2 (17)	6 (4)	9 (29)

*Not given in publication.

*NLRP3*-AID patients from both of large cohorts, the Eurofever Registry (the Eurofever group)^12^ and an international collaborative study (the CINCA group)^13^, were all Caucasians. Among them, all of the patients included in the CINCA group were of severe type of *NLRP3*-AID (CINCA)^13^, while the phenotypes of patients in the Eurofever group were not mentioned^12^. The incidences of eye involvement in our group (58%) and the Eurofever group (71%) were lower than that in the CINCA group (100%). The ocular manifestations in our study including conjunctivitis, uveitis, and glaucoma, were similar to those in the Eurofever group (58% vs. 64%, 17% vs. 7%, and 8% vs. 1%, respectively). The incidences of papilledema and severe manifestations such as optic neuritis and optic atrophy were higher in our group than those in the Eurofever group (42% vs. 21%, 17% vs. 0, and 17% vs. 4%, respectively). When compared to the CINCA group, uveitis, keratitis, vitritis, and cataract were less common (17% vs. 52%, 0 vs. 42%, 0 vs. 13%, and 0 vs. 16%, respectively), while papilledema and optic atrophy were similar in our cohort (42% vs. 42%, and 17% vs. 29%, respectively).

## Discussion

Because of the rarity and nonspecific symptoms of *NLRP3*-AID, diagnosis of this disease is easily delayed, as shown in our study, and the median time of diagnosis delay was 26 years (range 12–39). Although urticarial-like rash, arthralgia/arthritis, hearing loss and central nervous system involvement are the most common symptoms associated with fever, the ocular manifestations should not be neglected in *NLRP3*-AID. Literature has shown that ophthalmic findings could occur in all 3 types of *NLRP3*-AID, whereas prominently and critically in the severe type (CINCA)^13^. However, there is increasing evidence that patients with the moderate type (MWS) may experience similar ocular manifestations to CINCA^14–17^. Since all of the patients in our study were diagnosed as MWS, this is the first summarization of ocular manifestations in the moderate phenotype of *NLRP3*-AID in the Chinese population.

According to our study, the ophthalmic manifestations in Chinese adult patients with *NLRP3*-AID were not uncommon, with an incidence of up to 58%. A variety of ocular manifestations were found in our patients, including not only conjunctivitis, but also uveitis, papilledema, and serious ones such as optic neuritis and optic atrophy, which led to impaired vision or even blindness. Moreover, we found that patients with papilledema who were refractory to steroids and immunosuppressants had good responses to TNFα inhibitors (because IL-1 inhibitors are not available in China), and these improvements were usually accompanied by decrease of intracranial pressure, yet optic atrophy presented in some of them was irreversible. It indicates that ophthalmologists and rheumatologists should pay more attention to the eye involvement in *NLRP3*-AID patients, and regular ophthalmic examinations should be done, in order to make early and appropriate recognition and treatment.

Since the circulating levels of IL-1β and TNFα have increased prominently in *NLRP3*-AID patients^18^, biologics against these cytokines, especially IL-1 inhibitors work as therapeutic approaches. Nevertheless, some patients have inadequate responses^19^, and also, they are not available in China. In the meantime, TNFα inhibitors are second-line options for other monogenic autoinflammatory diseases such as TRAPS, MKD, and FMF^20^, therefore, they may be an alternative approach of *NLRP3*-AID. Previous studies have revealed that TNFα is an important transcriptional regulator of NLRP3 inflammasome components in murine inflammasomopathies, suggesting therapeutic implications for *NLRP3*-AID patients with inadequate responses to IL-1-targeted therapies^21^. Additionally, TNFα could promote the NLRP3 inflammasome activation through the NF-κB pathway, leading to caspase-1 activation and IL-1β secretion^22^. Taken these data together, we tried etanercept in 3 patients in our study, and all of them had good response.

It is noteworthy that nearly half of our patients had papilledema and one fifth had severe eye manifestations including optic neuritis and atrophy, which were more common than in the Caucasian patients^12^, suggesting that ethnicity and genotype may attribute to the differences of clinical characteristics. In addition, the incidences of papilledema and optic atrophy in our MWS patients were consistent with those in CINCA patients^13^. Although keratitis, vitritis and cataract were not seen in our patients, rare ocular manifestations such as anterior iris synechia, band keratopathy, and mild cataract were occasionally reported in the moderate type of *NLRP3*-AID^23^. Taken these data together, the moderate phenotype of *NLRP3*-AID (MWS) presents a diversity of ocular findings, which may be associated with the genotypes.

We have known that *NLRP3*-AID is caused by gain-of-function missense mutations of the *NLRP3* gene^24,25^. We identified five different variants in our *NLRP3*-AID patients, which were V70M, D303G, G326E, A439V and T348M. Previous studies have shown that the pathogenic variant T348M was presented in about 15% of individuals with *NLRP3*-AID^26^. It was associated with disease onset within six months of birth, a chronic course and hearing loss^27–32^. In our study, 3 out of 7 *NLRP3*-AID patients with ocular involvement carried T348M mutation. Indeed, they presented hearing loss and neurologic manifestations. Meanwhile, their eye manifestations were more serious than the other 4 patients, which included papilledema, optic neuritis, optic atrophy, and even blindness. Hence, we highly suspect that *NLRP3* T348M is related to severe ocular damage, though more research is needed in the future, especially in populations other than Chinese. A439V in exon 3 is also a pathogenic variant in *NLRP3*-AID. Interestingly, the first known patient of MWS with anterior uveitis described in 2007 by Shakeel and Gouws carried the A439V mutation^33^. Thereafter, some individual cases had been reported further demonstrating the relation of the A439V mutation and uveitis^14,34,35^. B. Sobolewska specifically focused on ocular symptoms in 37 members of a 5-generation family with the A439V mutation^36^, which was represented by conjunctivitis and anterior uveitis. Notably, patient 7 in our study carrying A439V variant also had a history of uveitis, although the current eye examinations of the patient showed no relevant findings. These data reinforce the genotype–phenotype relationship in *NLRP3*-AID. Pathogenic D303G variant which located in exon 3 was found in one patient in our study, who manifested as conjunctivitis, iritis, and papilledema. Moreover, we identified a *de novo* heterozygous V70M variant which located in exon 1 of *NLRP3* in patient 1. According to the Infevers database^28^, V70M is a variant with uncertain significance (VUS). Patient 1 had typical manifestations of periodic fever, urticarial-like rash, headache and conjunctivitis. In addition, since the minor allele frequency (MAF) of V70M was only 0.002 in Asian population, and it is predicted to be probably damaging when using an in silico analysis algorithm to predict the effects of this variant on protein function, we think it was probably pathogenic in this patient. G326E is a likely pathogenic variant of *NLRP3*, locating in exon 3. Its MAF was less than 0.0005 in Asian population. We also used an in silico analysis algorithm, and it is predicted to be probably damaging. Taken together, it is reasonable to consider the pathogenic effect of G326E in patient 6. To the best of our knowledge, there have been no reports about the associations between V70M, D303G, G326E and eye manifestations in *NLRP3*-AID. Further functional experiments are needed to clarify the pathogenic role of these VUS and likely pathogenic *NLRP3* variants identified in our study.

In conclusion, we reported the first cohort of adult *NLRP3*-AID patients with ocular involvement in the Chinese population. Ocular manifestations were diverse in *NLRP3*-AID, particularly in patients with the moderate phenotype (MWS), and may have relationship with genotypes. They could occur at the initial stage of *NLRP3*-AID, and were usually accompanied by skin, hearing and central nervous system involvements. Early recognition of these manifestations and prompt consultations with ophthalmologists could help to make early diagnosis and initiate appropriate therapy of this disease, and further avoid the irreversible ocular damages. Because of the limitation of patients’ number, further studies and follow-up of adult patients with *NLRP3*-AID in larger Chinese population will be necessary.
